# A domain-centric solution to functional genomics via dcGO *Predictor*

**DOI:** 10.1186/1471-2105-14-S3-S9

**Published:** 2013-02-28

**Authors:** Hai Fang, Julian Gough

**Affiliations:** 1 Department of Computer Science, University of Bristol, The Merchant Venturers Building, Bristol BS8 1UB, UK

## Abstract

**Background:**

Computational/manual annotations of protein functions are one of the first routes to making sense of a newly sequenced genome. Protein domain predictions form an essential part of this annotation process. This is due to the natural modularity of proteins with domains as structural, evolutionary and functional units. Sometimes two, three, or more adjacent domains (called supra-domains) are the operational unit responsible for a function, e.g. via a binding site at the interface. These supra-domains have contributed to functional diversification in higher organisms. Traditionally functional ontologies have been applied to individual proteins, rather than families of related domains and supra-domains. We expect, however, to some extent functional signals can be carried by protein domains and supra-domains, and consequently used in function prediction and functional genomics.

**Results:**

Here we present a domain-centric Gene Ontology (dcGO) perspective. We generalize a framework for automatically inferring ontological terms associated with domains and supra-domains from full-length sequence annotations. This general framework has been applied specifically to primary protein-level annotations from UniProtKB-GOA, generating GO term associations with SCOP domains and supra-domains. The resulting 'dcGO Predictor', can be used to provide functional annotation to protein sequences. The functional annotation of sequences in the Critical Assessment of Function Annotation (CAFA) has been used as a valuable opportunity to validate our method and to be assessed by the community. The functional annotation of all completely sequenced genomes has demonstrated the potential for domain-centric GO enrichment analysis to yield functional insights into newly sequenced or yet-to-be-annotated genomes. This generalized framework we have presented has also been applied to other domain classifications such as InterPro and Pfam, and other ontologies such as mammalian phenotype and disease ontology. The dcGO and its predictor are available at http://supfam.org/SUPERFAMILY/dcGO including an enrichment analysis tool.

**Conclusions:**

As functional units, domains offer a unique perspective on function prediction regardless of whether proteins are multi-domain or single-domain. The 'dcGO Predictor' holds great promise for contributing to a domain-centric functional understanding of genomes in the next generation sequencing era.

## Background

The first decade of this century has seen the rapid accumulation of vast genome-scale sequences, largely fuelled by the next generation sequencing technologies. Although these massive amounts of data offer an unprecedented opportunity for addressing many fundamental questions in the field of biomedical science [1,2], yet making sense of these raw sequences on their own represents a tremendous challenge. A large body of new protein sequences is awaiting functional annotations [3,4], which trails far behind by the rate of genome sequencing. Classically, sequence-function relationships for a protein are largely evident through looking at its structural properties. One of the most obvious structural properties for the protein is modular design, with domains forming distinct globular structural units. Apart from structural units, 3D domains are also evolutionarily related. For example, the Structural Classification of Proteins (SCOP) database [5] defines domains as the smallest unit of evolution. When it comes to function, however, we are accustomed to considering whole proteins despite the fact that very often domains can be functional units. As a matter of fact, domains can carry out many aspects of protein functions, and are widely used as functional predictors. Among current methods for computational protein function annotation/prediction [6,7], the structure-based methods are increasingly popular [8,9] as more structures are and will be resolved experimentally and deposited digitally in the Protein Data Bank (PDB) [10]. Without referring to detailed residual information of primary sequences, structural information at the domain level is closely relevant to biological functions. In principle, the coverage of functional annotations can be dramatically improved by *in silico *transferring known functions of proteins to those un-annotated proteins via their shared structures [11,12]. Hence, generating domain-centric functional annotations is necessary to realize such automated protein function transfer/prediction.

SCOP domains defined at *superfamily *and/or *family *levels are decent choices regarding the above-mentioned three aspects (structural, evolutionary and functional) of protein modularity [5]. At the *superfamily *(or evolutionary) level, domains are distantly related with evidence for common ancestry; within the same *superfamily*, domains are further divided into the *family *level wherein domains are often related by sequence similarity [13]. Based on SCOP, the SUPERFAMILY database uses hidden Markov models to detect and classify SCOP domains at both the *superfamily *and *family *levels [14]. Consequently, each protein sequence may be represented as a string of SCOP domains, called domain architectures [15]. To better understand the functional aspect of SCOP domains, recently we have also proposed a framework for automatically inferring the domain-centric annotations from the existing protein-level Gene Ontology (GO) annotations, and thereafter deriving a list of GO terms that are of most relevance to individual SCOP domains [16]. Although they are useful in describing functionally independent domains, most domains may not just function alone. When surveying domain compositions of proteins in the latest version of the UniProt Knowledgebase (UniProtKB) [17], we find that up to 70% are predicted to be multi-domain proteins. In multi-domain proteins, two or more domains can combine together, thus conferring functional plasticity. The recombination of the existing domains in multi-domain proteins is considered as one of driving forces for gaining functions (neo-functions or more complex functions) [18]. The combinations of two or more successive domains can be viewed as 'supra-domains' if they exist in different domain architectures [19]. In other words, supra-domain combinations can be found in different full-length domain architectures and act as larger evolutionary units greater in size than a single domain yet not necessarily a complete full-length protein architecture. Given the combinatorial nature of supra-domains, their functions are not practical to characterize in a labor-intensive manner. Supra-domains are far more difficult than individual domains to manually curate by looking at the functions of multi-domain proteins they reside in. Motivated by these challenges, additional research is warranted to explore how domain combinations contribute to function diversifications. It also remains to show whether the previously proposed framework can be extended to infer GO terms suitable for supra-domains in addition to individual domains. More importantly, there is a need to clarify the utility of GO-annotated domains and supra-domains in function prediction and other aspects of functional mining.

In an attempt to address the questions mentioned above, we first generalized our previous framework for capturing GO terms suitable for annotating both individual domains and supra-domains. Using the concept of reverse engineering, at the core of this domain-centric approach is: if a GO term tends to annotate a set of proteins containing a certain domain (or a set of proteins containing a supra-domain), then this term should also carry out functional signals for that domain (or supra-domain). Biologically, the resulting domain-centric GO (dcGO) annotations have carried on hidden functional signals buried under existing annotated proteins. Methodologically, this resource has taken into account the structural organization of GO by performing two types of statistical inference. Because of these considerations, a domain (supra-domain) can be associated with multiple GO terms (if any) that are informative to annotate it, and thus allowing multiple associations between domains and GO terms (quite similar to those between proteins and GO terms). Since the inferred dcGO can preserve the input information at the protein level, to some extent our approach addresses the challenges like one-domain-many-functions and one-function-many-domains (if there exists any evidence to support one2many associations). With the dcGO annotations at hand, we then developed 'dcGO Predictor' to predict functions of the target sequences in the CAFA experiment, an international competition for automatic protein function and critical assessment [20]. Finally, we derived meta-GO terms (GO slims) of different specificities, and showcased their related dcGO annotations to facilitate our understanding of functional implications in sequenced genomes. These results demonstrated the ability of the domain-centric solution towards function predictions and functional genomics.

## Results and discussion

### A domain-centric GO approach to automatically infer GO annotations for individual domains and supra-domains

**Figure 1:**
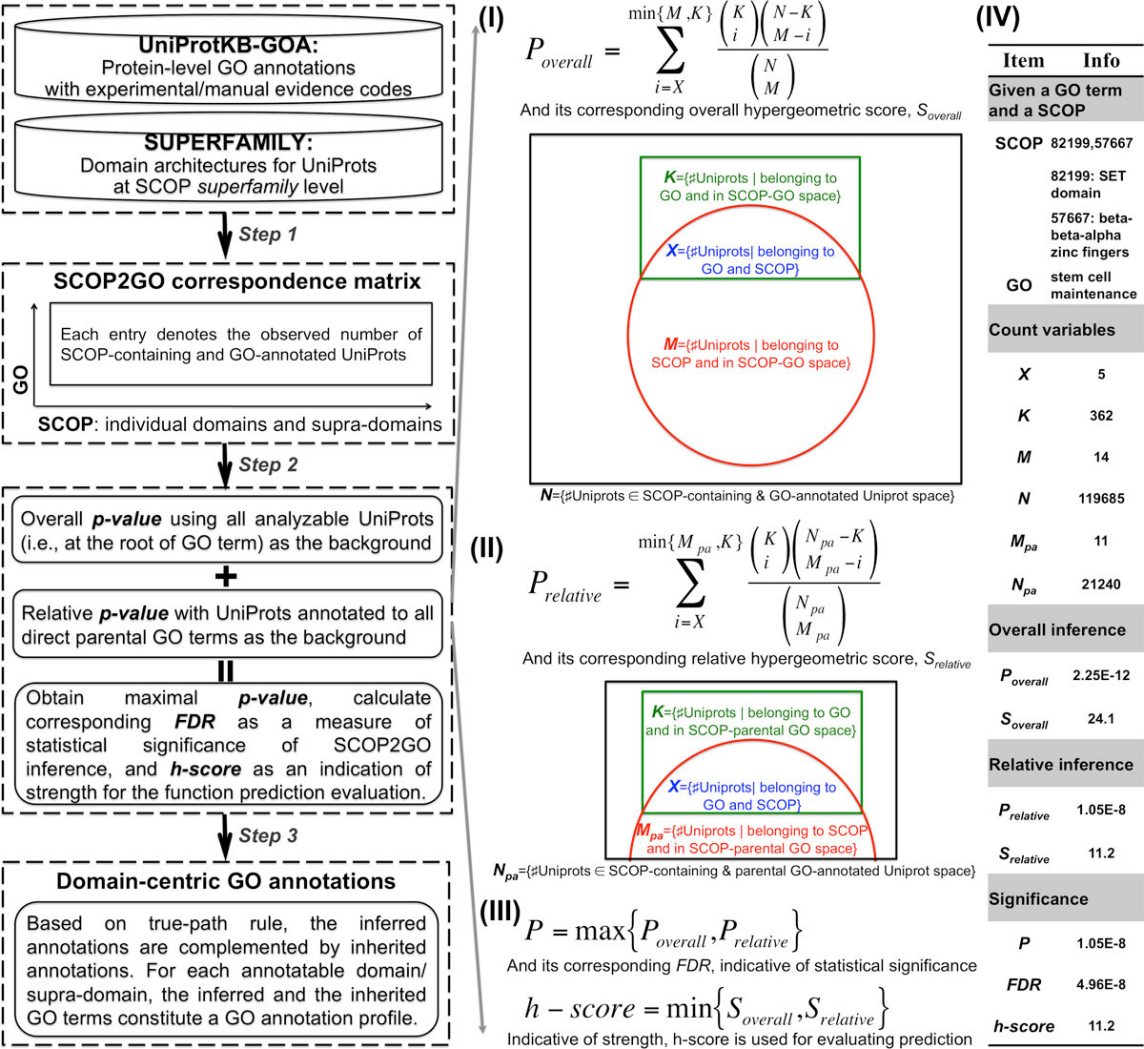
**A domain-centric GO approach to automatically infer GO annotations for individual domains and supra-domains**. The flowchart in the left panel illustrates three major steps of the proposed approach, including (*Step 1*) the preparation of the correspondence matrix between domains/supra-domains and GO terms from protein-level annotations in UniProtKB-GOA and domain architectures in SUPERFAMILY database, (*Step 2*) two types of statistical inference followed by FDR calculation, and (*Step 3*) following the true-path rule to obtain the complete domain-centric GO annotations. The overall inference (I), relative inference (II) and the significance measure (III) are illustrated in the middle panel, both mathematically and graphically. Further illustration (IV) is given by specifying an example of inferring associations between a supra-domain (i.e., '82199,57667') and a GO term (i.e., 'stem cell maintenance') in the right panel. Notably, there are a total of three direct parental GO terms (i.e., 'developmental process', 'negative regulation of cell differentiation', and 'stem cell development') for 'stem cell maintenance', and *N_pa _*is the total number of Uniprots that can be annotated by any direct parental GO terms.

The structural domain information of a protein is closely relevant to biological functions it has. To reveal the extent of functional signals carried by protein domains (and supra-domains in the multi-domain proteins), we developed a domain-centric Gene Ontology (dcGO) approach (Figure 1; see also Methods for details), a generalized extension to our previous proposal [16]. Briefly, the implementation of this approach started from high-coverage domain architectures and high-quality GO annotations for proteins (obtained respectively from SUPERFAMILY [21] and UniProKB-GOAs [22]), resulting in the correspondence matrix between domains/supra-domains and GO terms. Based on this matrix, two types of statistical inference (i.e., overall and relative inference) were performed while respecting the directed acyclic graph (DAG) of GO; these dual inferences aimed to ensure that only the most relevant GO terms could be retained. A false discovery rate (FDR) [23] was then calculated to measure significance of inference, while a hypergeometric score (h-score) used to indicate the strength of inference. Finally, we propagated the inferred GO terms to all their ancestors, generating the complete GO annotations for a domain/supra-domain. The middle panel in Figure 1 gives an account of analytic details, while the right panel illustrates an example of how to infer possible associations between a supra-domain '82199,57667' ('82199' stands for 'SET domain', and '57667' for 'beta-beta-alpha zinc fingers') and a GO term 'GO:0019827' ('stem cell maintenance'). The full results for this example are accessible at [24]. From this link and the Figure 1, we can see a significant association between the supra-domain and the GO term (FDR = 4.96E-8). Interestingly, among the two domains constituting this supra-domain, only 'SET domain' is associated with 'stem cell maintenance' (FDR = 7.15E-3; inherited annotation), but not for 'beta-beta-alpha zinc fingers'. This example clearly shows the necessity of associating two or longer supra-domains with GO terms, as functional units can consist of more than one domain acting together or acting at an interface between domains.

The resulting dcGO resource is available at [25], wherein the 'BROWSE' navigation on the left provides two options for accessing the resource. The first one is a SCOP-orientated option to obtain a list of GO terms annotated to individual domains (if annotatable), such as 'Triosephosphate isomerase (TIM)' [26]. The second option is a GO-orientated view to list those domains/supra-domains annotated by a GO term, like 'serine-type peptidase activity' [27] and 'stem cell maintenance' [24]. Such displays, if combined with a species tree of life (such as provided by SUPERFAMILY [28,29]), can greatly facilitate evolutionary analyses of either an individual domain of interest or sets of domains annotated by a GO term of interest.

Table 1 summarizes statistics in terms of annotatable domains/supra-domains, GO terms used, the total annotations observed, and the annotation density (defined as the ratio of the observed against the theoretical). Although annotatable supra-domains outnumber individual domains, the percentage of all possible supra-domains to be annotated is the lowest (27%~37%), compared to individual domains (46%~52% at SCOP *family *level and 64%~71% at SCOP *superfamily *level). When it comes to annotation density, however, these triple domain types share the same range for each of three GO ontologies, including Biological Process (BP, 0.008~0.013), Molecular Function (MF, 0.006~0.008) and Cellular Component (CC, 0.013~0.024). This un-skewed annotation density partially implies that dcGO approach has no biases towards processing individual domains and supra-domains. Although many more annotations will be inferred in the future as primary source databases improve, we do not expect the annotation density to change dramatically from one fortnight update to the next.

**Table 1:** A summary of statistics for domain-centric GO annotations.

	Ontology^1^	#Domains^2^	#Terms^3^	#Annotations^4^	#Density^5^
3,246 *FA *domains^6^	BP	1,696	6,699	106,855	9.41E-03
	MF	1,544	1,937	19,329	6.46E-03
	CC	1,490	902	30,084	2.24E-02
1,660 *SF *domains^7^	BP	1,177	8,632	127,680	1.26E-02
	MF	1,096	2,253	20,009	8.10E-03
	CC	1,054	1,134	28,636	2.40E-02
14,531 supra-domains^8^	BP	5,363	5,982	256,648	8.00E-03
	MF	3,972	1,472	33,355	5.70E-03
	CC	4,260	820	61,245	1.75E-02

^1^BP = Biological Process, MF = Molecular Function, CC = Cellular Component; ^2^The number of annotatable domains/supra-domains; ^3^The number of GO terms used to annotate; ^4^The total number of domain-centric GO annotations; ^5^Annotation density is defined as the observed (i.e., #Annotations) divided by the theoretical (a multiplication of #Domains and #Terms); ^6^A total of 3,246 distinct domains at SCOP *family *(*FA*) level; ^7^A total of 1,660 distinct domains at SCOP *superfamily *(*SF*) level; ^8^A total of 14,531 possible supra-domains.

### CAFA function prediction using GO annotations of both individual domains and supra-domains

To maximize performance, protein function prediction should integrate different kinds of predictive features [30-33], including the primary sequence for blast comparisons and non-sequence relevant features such as expression levels, sub-cellular localizations and protein-protein interactions, to name but a few examples. Rather than to train a multi-feature classifier or make consensus annotations, here we aim to show the contribution made to function prediction by the single-method dcGO direct annotations. Viewing domains as functional units renders the (organism independent) function prediction of poorly annotated proteins with known/predicted domains fairly straightforward. It was achieved via 'dcGO Predictor' [34]. The implementation first considers the domain composition of a target protein, and subsequently transfers any GO annotations of its residential domains/supra-domains to the target. The predictive score (p-score) is calculated to reflect the confidence of such predictions/transfers. We applied 'dcGO Predictor' to target sequences provided in the CAFA experiment (See the Methods section for details). Briefly, GO terms for MF and BP were predicted for these targets (7 eukaryotic sets and 11 prokaryotic sets), resulting in a list of terms along with a predictive score for each of targets. Then, these predictions were evaluated by the precision-recall (PR-RC) analysis against gold standard true annotations manually curated in a period of more than one year. Finally, all individual target PR-RC values were averaged to produce PR-RC values for each of the eukaryotic sets or the whole sets for eukaryote (and prokaryote).

**Figure 2:**
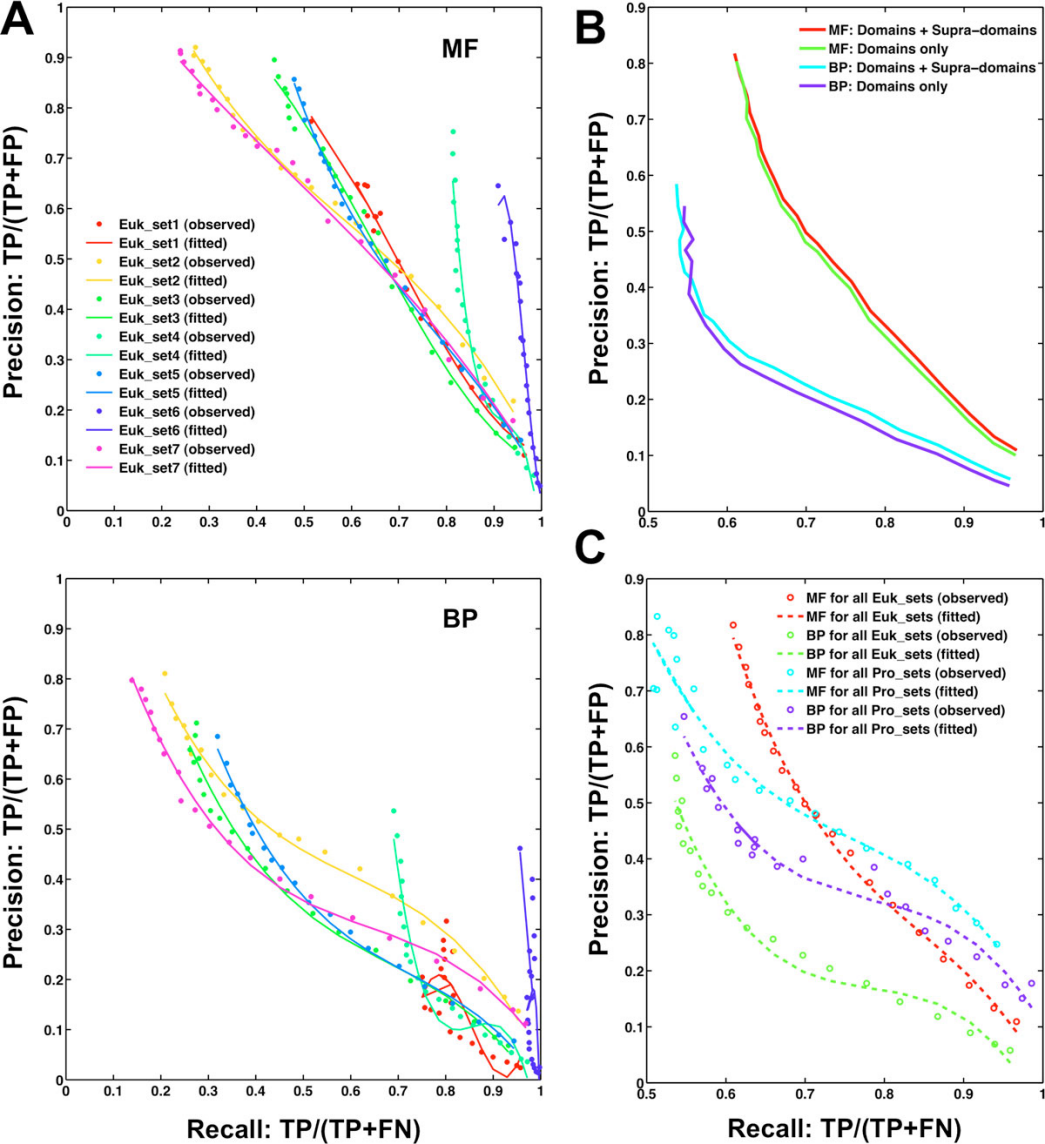
**The performance of dcGO Predictor in the CAFA experiment**. To evaluate function prediction, true prediction rate (precision: TP/[TP+FP]) and true positive rate (recall: TP/[TP+FN]), both as a function of the predictive score (see Methods) are plotted as a precision-recall (PR-RC) curve. **(A) **PR-RC curves for each of 7 sets of eukaryotic sequence targets, separately calculated using (*top panel*) the Molecular Function (MF) and (*bottom panel*) Biological Process (BP) ontologies. These predictions are based on GO annotations of both domains and supra-domains. **(B) **Comparisons of prediction using both domains and supra-domains against that using domains. PR-RC curves plotted here are for all eukaryotic sets as a whole. **(C) **Comparisons of PR-RC curves between eukaryotic sets and prokaryotic sets.

**Figure 3:**
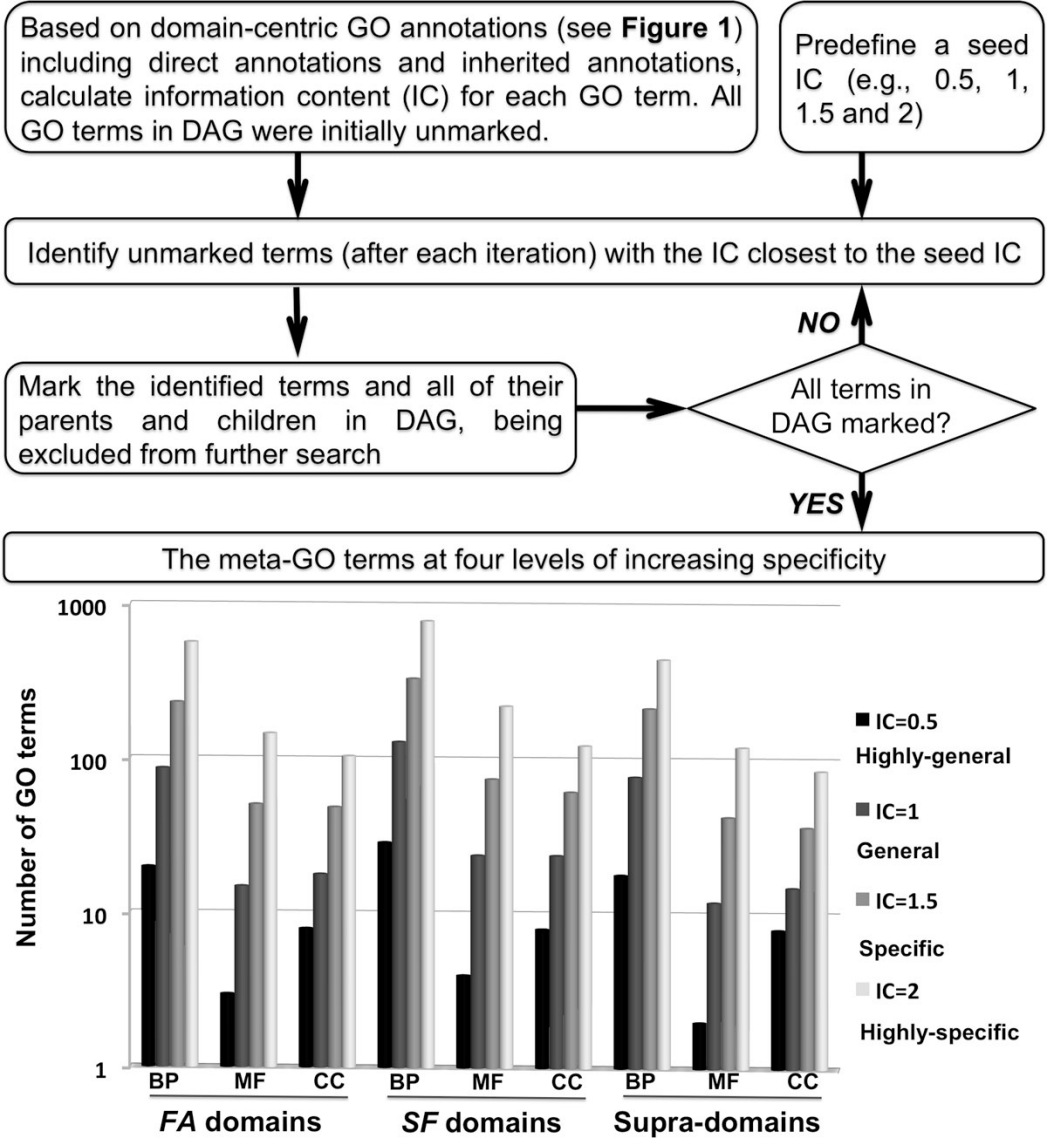
**Flow diagram of generating meta-GO terms through information content analysis of domain-centric GO annotations**. Briefly, all GO terms in DAG are initially unmarked. Then, identify those unmarked GO terms with IC closest to a predefined IC (e.g., 1). Mark those identified terms and all of their ancestors and descendants, being excluded from further search. Continue the previous two steps to iteratively identify unmarked GO terms until all GO terms in DAG are marked. Finally, output only those identified GO terms with IC falling in the range (e.g., [0.75 1.25]) as a meta-GO. The bottom panel displays the compositions in meta-GO terms for domains at SCOP *family *(*FA*) level, at SCOP *superfamily *(*SF*) level, and supra-domains.

We first examined the PR-RC curves of our prediction using both domains and supra-domains for eukaryotic sets (Figure 2A). Considering purely domain information is used, dcGO predictions were remarkably successful in recovering true functional annotations. Our prediction yielded the best results for Euk_set6, which is consistent with the highest percentages of annotatable domains/supra-domains. We also found that using GO terms in MF (top panel in Figure 2A) outperformed using those in BP (bottom panel in Figure 2A), indicating that molecular functional aspect is more relevant to describing the domains/supra-domains. Interestingly, limiting the prediction to the individual domains only slightly reduced performance when plotting PR-RC curves for the whole eukaryotic sets (Figure 2B). Further examination of domain compositions of these eukaryotic targets reveals that only one-third of the targets were of multi-domain proteins, which is far less than the average of 70% for eukaryotic proteins (as discussed in the Background section). We expect that the inclusion of supra-domains would lead to much better function prediction if a more representative set of multi-domain targets were to be included. When applied to prokaryotic sets (for which there is insufficient data for a proper evaluation, as stated in the CAFA experiment [20]), surprisingly we observed a similar overall performance to the eukaryotic sets (Figure 3C). This observation partially implies that the dcGO approach is not so sensitive to the sequences of different origins as long as these sequences to be predicted are not so atypical in terms of domain content they have.

'dcGO Predictor' is not just computationally efficient, but brings additional understanding to the annotation at a sub-protein resolution. Using modular domains is not just conceptually more intuitive, but easier to implement than other more complex methods. For example, we have extended the generalized domain-centric approach to other InterPro signatures [35]. The additional domains in InterPro further improve the predictive performance described above (Additional file 1). Notably, the revised version of 'dcGO Predictor' (as described here using h-scores to calculate p-scores) is more discriminative than the old version (used in CAFA before 15th, September 2010) that used the FDR to calculate p-scores. As shown in Additional file 2, the old version misses the 'higher precision but lower recall' part of PR-RC curves. This is because the FDR does not discriminate between high-scoring predictions (they all collapse to FDR = 0). Conversely, h-scores differ between the top predictions.

### The derivation of meta-GO terms and their application to functional genomic analyses

The 'dcGO Predictor' for function prediction in the CAFA experiment validates the quality of this dcGO annotations resource. To extend the usefulness of the resource, we generated meta-GO terms (Figure 3). Similar to the concept of GO slims [36], these meta-GO terms contain a subset of terms appearing in dcGO annotations but allow for a grain-specific view of the content. They were obtained by partitioning GO according to specificity measured in the form of information content (IC). As summarized in the bottom panel of Figure 3, meta-GO terms were divided into four levels of increasing specificity: highly general, general, specific, and highly specific. In agreement with the pyramid-like shape of the GO hierarchy, increasing specificity led to an increase in the number of GO terms in all cases. For a given ontology and a given specificity, we also noticed that there existed a similar number of GO terms, suggesting comparable compositions of meta-GO terms for individual domains and supra-domains.

Since the derived meta-GO terms provide a grain-specific overview, restricting their related dcGO annotations (rather than all of them) is particularly useful for GO term enrichment analysis. This kind of enrichment analysis is commonly employed for interpreting transcriptome data (such as by us [37,38] and others [39,40]), and can be generally applied to any large-scale dataset if provided with knowledge-specific annotations. To reveal the functional insights into newly sequenced or yet-to-be-annotated genomes, we viewed their domainome (a complete set of domains present thereof) as functional carriers and performed enrichment analysis using meta-GO terms and domain-centric annotations. So far, we have applied such functional genomic analysis to all sequenced eukaryote genomes provided by the SUPERFAMILY database. As a proof of principle, analysis of a recently sequenced and rapidly evolving animal lineage, *Oikopleura dioica *[41], showed that the enriched GO terms were diverse, representing a wide spectrum of functions involved in energy metabolism, organ/tissue development, responses to stress/stimulus, cell communication and signal transduction (Additional file 3). These functional implications are not only consistent with our previously identified over-represented domains (as compared to other eukaryotes) [42], but also provide a possible direction for future studies to clarify these observations in greater detail. In a second case, we analyzed three unicellular genomes (*Capsaspora owczarzaki, Monosiga brevicollis*, and *Proterospongia*). These genomes are phylogetically located at the animal-fungal boundary and thus afford an important look at the origin of the multicellular animals [43-46]. As listed in Additional file 4, these genomes shared quite a similar profile of GO enrichments, including metabolic processes, catalytic molecular function, and cellular organelle (particularly mitochondrion). Catalytic function and related metabolic processes appeared to a reasonable result of a large number of catalytic domains observed [45,47], which is becoming the focus of considerable research [48,49]. The functional involvement of mitochondrion was probably reflective of the importance of mitochondrial genomes in putting themselves as an out-group to animals [50]. In the near future, we will extend these functional genomic analyses in a context of a species tree to reveal functional trends in the course of eukaryotic evolution. Other than the genomic data stored in SUPERFAMILY, user-submitted domains are also supported for enrichment analysis. This 'dcGO Enrichment' [51] can provide the interpretations for a predefined list of domains of interest, for example, a list of unusual domains found in a genome as compared to other genomes.

## Conclusions

In this work, we present a domain-centric GO approach of using protein-level annotations and structural information to detect functional signals inherent in the residential domains/supra-domains. Under this principle we have developed 'dcGO Predictor' as a functional annotation tool and we demonstrate its utility for protein function prediction in the CAFA experiment. Since 'dcGO Predictor' was not conceived as a competitor for the many multi-feature classifiers trained to perform on the CAFA test, its relative success as a single direct method justifies its unique value. The generality of the method has allowed us to include many other biomedical ontologies in addition to GO, and allows it to be adopted more widely by other domain databases such as those in the InterPro consortium.

Using as a functional annotation tool, we demonstrate its utility for protein function prediction in the CAFA experiment, and this kind of domain-centric functional information should be incorporated into any future studies of genome annotations. We also generate meta-GO terms of different specificities and showcase their use for domain-based functional genomics. These results have already provided us and others (such as [52]) with a resource to understand the > 80 million (at the time of writing) sequences, both structurally and functionally.

## Methods

### Protein-level GO annotations in the UniProtKB-GOA database

GO annotations for proteins over a wide spectrum of species (~2,000) were downloaded from the UniProtKB on 19th, November 2011 [22]. Only those annotations using experimental or manual evidence codes [53] were retrieved so as to minimize the false-positives as training input. In total, there remained more than 157,000 proteins with at least a high-quality GO annotation. Almost half of annotations to these proteins were supported by the two top evidence codes, either 'IDA' (i.e., direct assay) or 'IMP' (i.e., mutant phenotype).

### Protein domain assignments in the SUPERFAMILY database

In the SUPERFAMILY database [16], protein domain assignments for UniProt proteins are monthly updated. It is done automatically using the HMMER3 package [54] and expert-curated hidden Markov models representing all protein domains of known structure [14]. Among GO-annotated proteins in UniProt, two thirds (over 100,000) were assigned to 1,660 distinct domains at SCOP *superfamily *(*SF*) level and 3,246 distinct domains at SCOP *family *(*FA*) level. A vast number of analyzable UniProt protein space (i.e., over 100,000 proteins with GO annotations and domain assignments) secures the adequate power of statistical inference carried out in this study.

### The definition of supra-domains and being available from the SUPERFAMILY database

In multidomain proteins, a certain domain tends to co-occur/co-evolve with other domains. Considering such promiscuous nature [55] and following on from our previous descriptions [19], we defined combinations of two or more successive domains as 'supra-domains' if such combinations were found in more than one distinct domain architecture. The domain architecture is a modular view of a protein sequence; in the SUPERFAMILY database, it is represented as the sequential order of SCOP domains (at the *superfamily *level) or gaps (estimated to be one or more unknown domains). The SUPERFAMILY database contains a total of 14,531 fully-annotated supra-domains (i.e. containing no unknown domains) that are present in analyzable UniProt protein space.

### Statistical inference for GO annotations of individual domains and supra-domains

The brief summary is illustrated in Figure 1. Along with it, we go through each step in greater detail in the rest of this section.

#### Two data sources

We took UniProt proteins with experimentally or manually curated GO annotations and high-coverage domain/supra-domain assignments as two training inputs. The correspondence matrix between domains/supra-domains and GO terms are tabulated with each entry as observed number of Uniprot proteins which contain that domain/supra-domain (given in column) and can be annotated by that GO term (given in row).

#### Two statistical inferences

We used the hypergeometric distribution as a null-hypothesis and performed statistical test (an equivalent to Fisher's exact test) to infer the possible associations between a GO term and a domain/supra-domain. Terms in GO are not isolated; rather they are organized as a directed acyclic graph (DAG) by viewing individual terms as a node and its relations to parental terms (allowing for multiple parents) as directed edges. Moreover, GO follows 'true-path rule', that is, a protein/domain annotated to a term should also be annotated by its all parent terms. To respect DAG structure and true-path rule, we conducted two types of statistical inferences. First, we calculated an overall p-value (and the corresponding overall hypergeometric score, that is, standard score or z-score, which is calculated by the observed minus the expected and then divided by standard deviation under hypergeometric distribution) using all analyzable UniProt proteins (i.e., those annotated to the root of GO term after applying the true-path rule) as the background. We also calculated a relative p-value (and the corresponding relative hypergeometric score) using the background of only those UniProt proteins annotated to all direct parental GO terms.

#### Significance and strength of associations

We first took the larger one of the overall and relative p-values from (2) to indicate the likelihood of associations between that GO term and that domain/supra-domain. To account for the multiple hypothesis testing, the Benjamini-Hochberg derived FDR [23] rather than the p-value was used to determine the statistical significance of associations between domains/supra-domains and GO terms. A stringent threshold of FDR (< 10^-3^) was accepted to statistically infer GO annotations of individual domains and supra-domains. In addition to FDR as significant measure, we also took the smaller of the overall and relative hypergeometric scores from (2) to indicate the strength of associations, denoted as h-score.

#### Direct and inherited annotations

According to the true-path rule, the inferred GO terms for a domain/supra-domain were propagated to all ancestor terms, along with the FDR and h-score (that is, the minimum FDR and the maximum h-score among all descendants if an ancestor term has multiple descendant terms annotating that domain/supra-domain). The inferred originally were called as direct annotations, the propagated as inherited annotations; both of them constituted a GO annotation profile in DAG. Notably, each annotation was associated with the FDR (indicative of statistical significant, and being less than < 10^-3^) and the h-score (indicative of strength, and the higher the stronger association). The latter was used for the evaluation of protein function prediction.

### Function prediction of target sequences from the CAFA experiment

The CAFA experiment [20] provided nearly 47,000 protein sequences for function prediction, including 7 eukaryotic sets and 11 prokaryotic sets. These targets were not annotated using 'EXP', 'TAS' or 'IC' evidence codes when available on the submission deadline (15th, September 2010). The added-in annotations under these evidence codes thereafter (till the evaluation time on 19th, November 2011) were served as gold positive standards for evaluating the function prediction. Both of the prediction and evaluation were restricted to MF and BP ontologies. Since the prokaryotic sets were exploratory only (insufficient for evaluation), eukaryotic sets were mainly focused on for the prediction and evaluation.

For the prediction part (i.e., 'dcGO Predictor' [34]), we first generated domain architectures for the targets and their derived domains and supra-domains. Then, the domain-centric GO inferred above was used for function prediction. If a target contained a domain/supra-domain, then all GO terms associated to that domain/supra-domain were transferred to the target (together with h-scores). When a target-to-term transfer was supported by one or more residential domains/supra-domains, we calculated a predictive score (p-score) by additively summing up h-scores and being scaled to the range of 0-1 (see **Eq. 1**). The higher value of the p-score indicates the more evident the prediction is. Each target (if predictable) was accompanied by a list of GO terms along with the corresponding predictive scores. The intuition behind this simple calculation is to rank the predictive p-scores for precision-recall analysis below.

(1) p-score=(SUM-MIN)/(MAX-MIN),

where ***SUM ***is the sum of all h-scores to support a GO term transferred to the target, ***MIN ***and ***MAX ***are respectively the minimum and maximum of ***SUM ***over a whole list of predicted GO terms for the target.

For the evaluation part, we first obtained a total of about 246,000 annotations newly added until 19th, November 2011. Based on these gold standards, then we estimated precision (PR) and recall (RC) for each sequence target under a given p-score (say ***t***) using **Eq. 2 **and **Eq. 3**. All these calculations were done separately for GO terms in BP and GO terms in MF. From individual target-specific PR vs. RC values, the precision (and recall) of each of eukaryotic sets (and the whole sets) was further calculated as an average over all targets contained in each set (and the whole sets).

(2) PR=TP/(TP+FP),

(3) RC=TP/(TP+FN),

where ***TP ***is true positives - calculated as the number of the predicted GO terms (with p-score larger than ***t***) overlapped with gold standards, ***FP ***for false positives - the number of the predicted GO terms that are not in gold standards, ***FN ***for false negatives - the number of gold standards that are not in the predicted GO terms.

### Deriving meta-GO terms of different specificities for domain-based GO enrichment analysis

We used information content (IC) of a GO term to measure its specificity in meaning of individual domain and supra-domain annotations (directed and inherited). For a given GO term, we defined IC as negative 10-based log-transformed frequency of domains/supra-domains annotated to that term. The reason behind using IC rather than the GO tree-like structure is that the GO was originally designed for annotating proteins, and some parts of GO structure might be irrelevant to annotate domains/supra-domains. Similar to our previous report [16], a search procedure was applied to iteratively walk every possible path in DAG for partitioning GO under a seed IC. Each partition was reflective of certain same specificity and contained GO terms located in distinct paths. Four levels of increasing granularity were defined, that is, being highly general, general, specific, and highly specific. Based on these meta-GO terms and their domain-centric annotations, we performed enrichment analysis of domainome assigned to sequenced eukaryotic genomes (stored in the SUPERFAMILY database). Enrichment analysis was based on the hypergeometric distribution, followed by FDR-based assessment of the statistical significance of GO enrichments [23]. Like 'dcGO Predictor', the 'dcGO Enrichment' [51] is also available to identify functions and other higher-order knowledge enriched within a list of protein domains that are submitted by the user.

### Data Availability

In additional to two SCOP-orientated and GO-orientated options for the navigations (the most-left 'BROWSE'), we also provided flat files and MySQL tables for the download on the dcGO website [56].

## Abbreviations

BP: Biological Process; CC: Cellular Component; dcGO: domain-centric Gene Ontology; DAG: directed acyclic graph; FDR: false discovery rate; FN: false negatives; FP: false positives; IC: information content; MF: Molecular Function; PR: precision; RC: recall; FA: SCOP family; SF: SCOP superfamily; TP: true positives; UniProtKB: UniProt Knowledgebase.

## Competing interests

The authors declare that they have no competing interests.

## Authors' contributions

HF conceived and designed the study, performed the data analysis and interpretation, and wrote manuscript. JG conceived and coordinated the study, contributed to data interpretations and manuscript preparation. Both authors read and approved the final manuscript.

## Supplementary Material

Additional file 1 **CAFA function prediction using SCOP individual domains and supra-domains plus InterPro domains. (A) **Precision-recall curves based on GO annotations of both domains and supra-domains. The left panel is for the Molecular Function (MF), and the right panel for Biological Process (BP). **(B) **The same as in **(A) **but using additional InterPro domains (excluding SCOP *superfamily *domains, SF). Click here for file

Additional file 2 **Performance comparisons between the currently revised version of 'dcGO Predictor' and the old version**. The revised version uses h-scores to calculate p-score while the old version (originally involved in CAFA before 15th, September 2010) uses the FDR to calculate p-scores. Click here for file

Additional file 3 **Enriched GO terms for domain repertoire present at a rapidly evolving metazoan, *Oikopleura dioica***. Click here for file

Additional file 4 **Enriched GO terms for domain repertoire present at each of three genomes of animal-fungal boundary**. Click here for file

## Declarations

This article has been published as part of *BMC Bioinformatics *Volume 14 Supplement 3, 2013: Proceedings of Automated Function Prediction SIG 2011 featuring the CAFA Challenge: Critical Assessment of Function Annotations. The full contents of the supplement are available online at http://www.biomedcentral.com/bmcbioinformatics/supplements/14/S3.

## References

[B1] Metzker ML Sequencing technologies - the next generation Nat Rev Genet 2010 11 1 31 46 10.1038/nrg2626 19997069

[B2] Ledford H Big science: The cancer genome challenge Nature 2010 464 7291 972 974 10.1038/464972a 20393534

[B3] Reeves GA Talavera D Thornton JM Genome and proteome annotation: organization, interpretation and integration J R Soc Interface 2009 6 31 129 147 10.1098/rsif.2008.0341 19019817 PMC2658791

[B4] Hawkins T Chitale M Kihara D New paradigm in protein function prediction for large scale omics analysis Mol Biosyst 2008 4 3 223 231 10.1039/b718229e 18437265

[B5] Andreeva A Howorth D Chandonia JM Brenner SE Hubbard TJ Chothia C Murzin AG Data growth and its impact on the SCOP database: new developments Nucleic Acids Res 2008 36 Database D419 425 18000004 10.1093/nar/gkm993 PMC2238974

[B6] Rentzsch R Orengo CA Protein function prediction--the power of multiplicity Trends Biotechnol 2009 27 4 210 219 10.1016/j.tibtech.2009.01.002 19251332

[B7] Friedberg I Automated protein function prediction--the genomic challenge Brief Bioinform 2006 7 3 225 242 10.1093/bib/bbl004 16772267

[B8] Malmstrom L Riffle M Strauss CE Chivian D Davis TN Bonneau R Baker D Superfamily assignments for the yeast proteome through integration of structure prediction with the gene ontology PLoS Biol 2007 5 4 e76 10.1371/journal.pbio.0050076 17373854 PMC1828141

[B9] Drew K Winters P Butterfoss GL Berstis V Uplinger K Armstrong J Riffle M Schweighofer E Bovermann B Goodlett DR The Proteome Folding Project: proteome-scale prediction of structure and function Genome Res 2011 21 11 1981 1994 10.1101/gr.121475.111 21824995 PMC3205581

[B10] Velankar S Best C Beuth B Boutselakis CH Cobley N Sousa Da Silva AW Dimitropoulos D Golovin A Hirshberg M John M PDBe: Protein Data Bank in Europe Nucleic Acids Res 2010 38 Database D308 317 10.1093/nar/gkp916 19858099 PMC2808887

[B11] Punta M Ofran Y The rough guide to in silico function prediction, or how to use sequence and structure information to predict protein function PLoS Comput Biol 2008 4 10 e1000160 10.1371/journal.pcbi.1000160 18974821 PMC2518264

[B12] Lee D Redfern O Orengo C Predicting protein function from sequence and structure Nat Rev Mol Cell Biol 2007 8 12 995 1005 10.1038/nrm2281 18037900

[B13] Madera M Vogel C Kummerfeld SK Chothia C Gough J The SUPERFAMILY database in 2004: additions and improvements Nucleic Acids Res 2004 32 Database D235 239 14681402 10.1093/nar/gkh117 PMC308851

[B14] Gough J Karplus K Hughey R Chothia C Assignment of homology to genome sequences using a library of hidden Markov models that represent all proteins of known structure J Mol Biol 2001 313 4 903 919 10.1006/jmbi.2001.5080 11697912

[B15] Wilson D Madera M Vogel C Chothia C Gough J The SUPERFAMILY database in 2007: families and functions Nucleic Acids Res 2007 35 Database D308 313 10.1093/nar/gkl910 17098927 PMC1669749

[B16] de Lima Morais DA Fang H Rackham OJ Wilson D Pethica R Chothia C Gough J SUPERFAMILY 1.75 including a domain-centric gene ontology method Nucleic Acids Res 39 Database D427 434 21062816 10.1093/nar/gkq1130 PMC3013712

[B17] Ongoing and future developments at the Universal Protein Resource Nucleic Acids Res 2012 39 Database D214 219 10.1093/nar/gkq1020 PMC3013648 21051339

[B18] Chothia C Gough J Genomic and structural aspects of protein evolution Biochem J 2009 419 1 15 28 10.1042/BJ20090122 19272021

[B19] Vogel C Berzuini C Bashton M Gough J Teichmann SA Supra-domains: evolutionary units larger than single protein domains J Mol Biol 2004 336 3 809 823 10.1016/j.jmb.2003.12.026 15095989

[B20] Automated Function Prediction: Critical Assessment of Function Annotations (CAFA) http://biofunctionprediction.org

[B21] SUPERFAMILY database of sturctural and functional protein annotatioins for all completely sequenced organisms http://supfam.org

[B22] Gene Ontology Annotation (UniProt-GOA) Database http://www.ebi.ac.uk/GOA/

[B23] Benjamini Y Hochberg Y Controlling the False Discovery Rate - a Practical and Powerful Approach to Multiple Testing Journal of the Royal Statistical Society Series B-Methodological 1995 57 1 289 300

[B24] Biological Process (BP): stem cell maintenance http://supfam.org/SUPERFAMILY/cgi-bin/dcgo.cgi?go=0019827

[B25] Fang H Gough J dcGO: database of domain-centric ontologies on functions, phenotypes, diseases and more http://supfam.org/SUPERFAMILY/dcGO/10.1093/nar/gks1080 PMC3531119 23161684

[B26] Triosephosphate isomerase (TIM) superfamily http://supfam.org/SUPERFAMILY/cgi-bin/dcscop.cgi?sunid=51351

[B27] Molecular Function (MF): serine-type peptidase activity http://supfam.org/SUPERFAMILY/cgi-bin/dcgo.cgi?go=0008236

[B28] Wilson D Pethica R Zhou Y Talbot C Vogel C Madera M Chothia C Gough J SUPERFAMILY--sophisticated comparative genomics, data mining, visualization and phylogeny Nucleic Acids Res 2009 37 Database D380 386 10.1093/nar/gkn762 19036790 PMC2686452

[B29] Pethica R Barker G Kovacs T Gough J TreeVector: scalable, interactive, phylogenetic trees for the web PLoS One 2010 5 1 e8934 10.1371/journal.pone.0008934 20126613 PMC2812488

[B30] Pena-Castillo L Tasan M Myers CL Lee H Joshi T Zhang C Guan Y Leone M Pagnani A Kim WK A critical assessment of Mus musculus gene function prediction using integrated genomic evidence Genome Biol 2008 9 Suppl 1 S2 10.1186/gb-2008-9-s1-s2 18613946 PMC2447536

[B31] Kourmpetis YA van Dijk AD van Ham RC ter Braak CJ Genome-wide computational function prediction of Arabidopsis proteins by integration of multiple data sources Plant Physiol 2011 155 1 271 281 10.1104/pp.110.162164 21098674 PMC3075770

[B32] Troyanskaya OG Dolinski K Owen AB Altman RB Botstein D A Bayesian framework for combining heterogeneous data sources for gene function prediction (in Saccharomyces cerevisiae) Proc Natl Acad Sci USA 2003 100 14 8348 8353 10.1073/pnas.0832373100 12826619 PMC166232

[B33] Nariai N Kolaczyk ED Kasif S Probabilistic protein function prediction from heterogeneous genome-wide data PLoS One 2007 2 3 e337 10.1371/journal.pone.0000337 17396164 PMC1828618

[B34] dcGO Predictor http://supfam.org/SUPERFAMILY/cgi-bin/dcpredictormain.cgi

[B35] Hunter S Jones P Mitchell A Apweiler R Attwood TK Bateman A Bernard T Binns D Bork P Burge S InterPro in 2011: new developments in the family and domain prediction database Nucleic Acids Res 2012 40 D1 D306 D312 10.1093/nar/gkr948 22096229 PMC3245097

[B36] Davis MJ Sehgal MS Ragan MA Automatic, context-specific generation of Gene Ontology slims BMC Bioinformatics 2010 11 498 10.1186/1471-2105-11-498 20929524 PMC3098080

[B37] Fang H Wang K Zhang J Transcriptome and proteome analyses of drug interactions with natural products Curr Drug Metab 2008 9 10 1038 1048 10.2174/138920008786927802 19075620

[B38] Fang H Yang Y Li C Fu S Yang Z Jin G Wang K Zhang J Jin Y Transcriptome analysis of early organogenesis in human embryos Dev Cell 2010 19 1 174 184 10.1016/j.devcel.2010.06.014 20643359

[B39] Subramanian A Tamayo P Mootha VK Mukherjee S Ebert BL Gillette MA Paulovich A Pomeroy SL Golub TR Lander ES Gene set enrichment analysis: a knowledge-based approach for interpreting genome-wide expression profiles Proc Natl Acad Sci USA 2005 102 43 15545 15550 10.1073/pnas.0506580102 16199517 PMC1239896

[B40] Sherman BT Huang da W Tan Q Guo Y Bour S Liu D Stephens R Baseler MW Lane HC Lempicki RA DAVID Knowledgebase: a gene-centered database integrating heterogeneous gene annotation resources to facilitate high-throughput gene functional analysis BMC Bioinformatics 2007 8 426 10.1186/1471-2105-8-426 17980028 PMC2186358

[B41] Denoeud F Henriet S Mungpakdee S Aury JM Da Silva C Brinkmann H Mikhaleva J Olsen LC Jubin C Canestro C Plasticity of animal genome architecture unmasked by rapid evolution of a pelagic tunicate Science 2010 330 6009 1381 1385 10.1126/science.1194167 21097902 PMC3760481

[B42] Chavali S Morais DA Gough J Babu MM Evolution of eukaryotic genome architecture: Insights from the study of a rapidly evolving metazoan, Oikopleura dioica: Non-adaptive forces such as elevated mutation rates may influence the evolution of genome architecture Bioessays 2011 33 8 592 601 10.1002/bies.201100034 21681984

[B43] Michod RE Evolution of individuality during the transition from unicellular to multicellular life Proc Natl Acad Sci USA 2007 104 Suppl 1 8613 8618 17494748 10.1073/pnas.0701489104 PMC1876437

[B44] Sebe-Pedros A de Mendoza A Lang BF Degnan BM Ruiz-Trillo I Unexpected repertoire of metazoan transcription factors in the unicellular holozoan Capsaspora owczarzaki Mol Biol Evol 2011 28 3 1241 1254 10.1093/molbev/msq309 21087945 PMC4342549

[B45] King N Westbrook MJ Young SL Kuo A Abedin M Chapman J Fairclough S Hellsten U Isogai Y Letunic I The genome of the choanoflagellate Monosiga brevicollis and the origin of metazoans Nature 2008 451 7180 783 788 10.1038/nature06617 18273011 PMC2562698

[B46] Ruiz-Trillo I Burger G Holland PW King N Lang BF Roger AJ Gray MW The origins of multicellularity: a multi-taxon genome initiative Trends Genet 2007 23 3 113 118 10.1016/j.tig.2007.01.005 17275133

[B47] Manning G Young SL Miller WT Zhai Y The protist, Monosiga brevicollis, has a tyrosine kinase signaling network more elaborate and diverse than found in any known metazoan Proc Natl Acad Sci USA 2008 105 28 9674 9679 10.1073/pnas.0801314105 18621719 PMC2453073

[B48] Conejo M Bertin M Pomponi SA Ellington WR The early evolution of the phosphagen kinases--insights from choanoflagellate and poriferan arginine kinases J Mol Evol 2008 66 1 11 20 10.1007/s00239-007-9058-0 18064398

[B49] Lim WA Pawson T Phosphotyrosine signaling: evolving a new cellular communication system Cell 2010 142 5 661 667 10.1016/j.cell.2010.08.023 20813250 PMC2950826

[B50] Lavrov DV Key transitions in animal evolution: a mitochondrial DNA perspective Integr Comp Biol 2007 47 5 734 743 10.1093/icb/icm045 21669754

[B51] dcGO Enrichment http://supfam.org/SUPERFAMILY/cgi-bin/dcenrichment.cgi

[B52] Parikesit AA Stadler PF Prohaska SJ Evolution and Quantitative Comparison of Genome-Wide Protein Domain Distributions Genes 2011 2 4 912 924 10.3390/genes2040912 PMC3927604 24710298

[B53] Rogers MF Ben-Hur A The use of gene ontology evidence codes in preventing classifier assessment bias Bioinformatics 2009 25 9 1173 1177 10.1093/bioinformatics/btp122 19254922

[B54] Eddy SR A new generation of homology search tools based on probabilistic inference Genome Inform 2009 23 1 205 211 20180275

[B55] Basu MK Carmel L Rogozin IB Koonin EV Evolution of protein domain promiscuity in eukaryotes Genome Res 2008 18 3 449 461 10.1101/gr.6943508 18230802 PMC2259109

[B56] Fang H Gough J dcGO: database of domain-centric ontologies on functions, phenotypes, diseases and more Nucleic Acids Res 2013 41 D536 544 23161684 10.1093/nar/gks1080 PMC3531119

