# Supplementary material for: A domain-centric solution to functional genomics via dcGO Predictor
Source: BMC Bioinformatics. 2013 Feb 28;14(Suppl 3):S9. doi: 10.1186/1471-2105-14-S3-S9 (PMC3584936; doi:10.1186/1471-2105-14-S3-S9)
Supplement: Additional file 1 — CAFA function prediction using SCOP individual domains and supra-domains plus InterPro domains. (A) Precision-recall curves based on GO annotations of both domains and supra-domains. The left panel is for the Molecular Function (MF), and the right panel for Biological Process (BP). (B) The same as in (A) but using additional InterPro domains (excluding SCOP superfamily domains, SF). [file 1471-2105-14-S3-S9-S1.pdf]

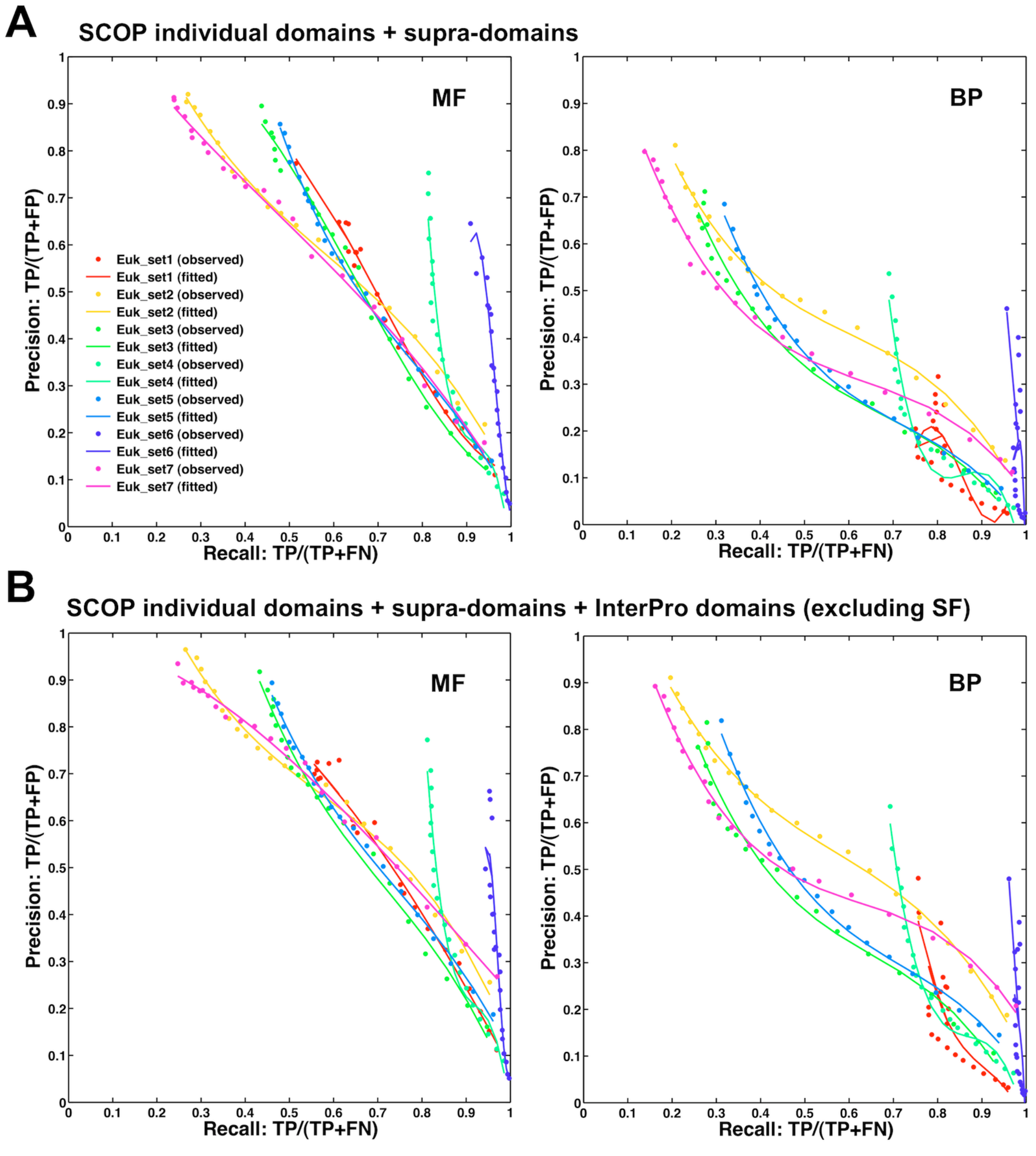

A
SCOP individual domains + supra-domains
MF
BP
Precision: TP/(TP+FP)
Recall: TP/(TP+FN)
Euk_set1 (observed)
Euk_set1 (fitted)
Euk_set2 (observed)
Euk_set2 (fitted)
Euk_set3 (observed)
Euk_set3 (fitted)
Euk_set4 (observed)
Euk_set4 (fitted)
Euk_set5 (observed)
Euk_set5 (fitted)
Euk_set6 (observed)
Euk_set6 (fitted)
Euk_set7 (observed)
Euk_set7 (fitted)
B
SCOP individual domains + supra-domains + InterPro domains (excluding SF)
MF
BP
Precision: TP/(TP+FP)
Recall: TP/(TP+FN)
