# Supplementary figures and images for: A domain-centric solution to functional genomics via dcGO Predictor
Source: BMC Bioinformatics. 2013 Feb 28;14(Suppl 3):S9. doi: 10.1186/1471-2105-14-S3-S9 (PMC3584936; doi:10.1186/1471-2105-14-S3-S9)

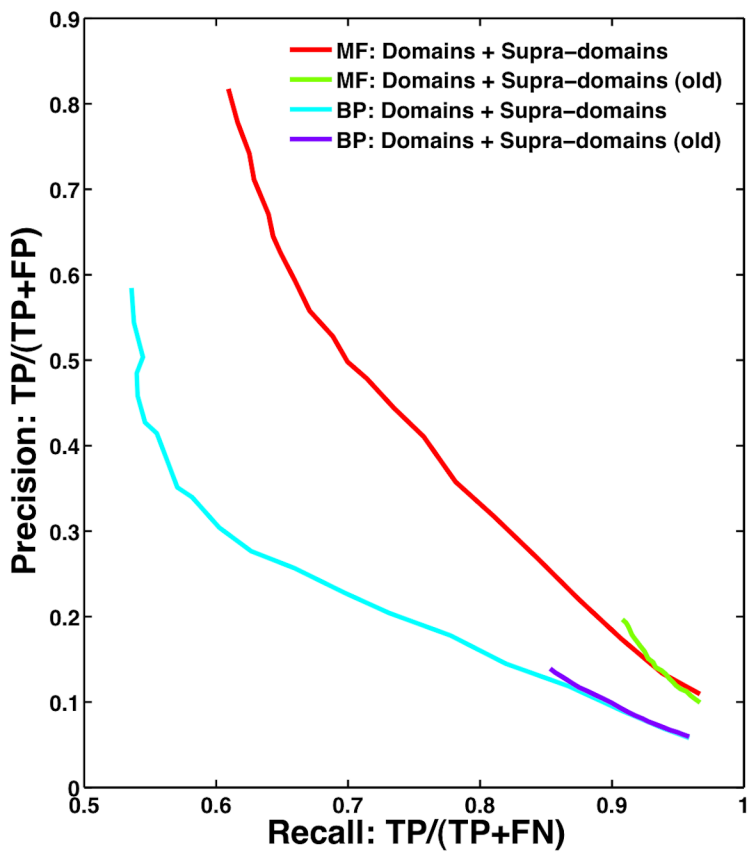

Supplement: Additional file 2 — Performance comparisons between the currently revised version of 'dcGO Predictor' and the old version. The revised version uses h-scores to calculate p-score while the old version (originally involved in CAFA before 15th, September 2010) uses the FDR to calculate p-scores. [file 1471-2105-14-S3-S9-S2.pdf]
