# Supplementary material for: A domain-centric solution to functional genomics via dcGO Predictor
Source: BMC Bioinformatics. 2013 Feb 28;14(Suppl 3):S9. doi: 10.1186/1471-2105-14-S3-S9 (PMC3584936; doi:10.1186/1471-2105-14-S3-S9)
Supplement: Additional file 3 — Enriched GO terms for domain repertoire present at a rapidly evolving metazoan, Oikopleura dioica. [file 1471-2105-14-S3-S9-S3.pdf]

**Additional file 3. Enriched GO terms for domain repertoire present at a rapidly evolving metazoan, *Oikopleura dioica*.**

| Ontology | Level | GO Term | FDR |
|---|---|---|---|
| BP | Highly-general | primary metabolic process | 1.50E-10 |
| | | cellular component organization at cellular level | 3.71E-07 |
| | | cellular component biogenesis | 7.62E-06 |
| | | regulation of biological quality | 4.02E-05 |
| | | organ development | 4.92E-05 |
| | | regulation of metabolic process | 9.99E-05 |
| | | nitrogen compound metabolic process | 1.37E-04 |
| | | cellular response to stimulus | 1.66E-04 |
| | | small molecule metabolic process | 1.76E-04 |
| | | anatomical structure morphogenesis | 4.30E-04 |
| | | biosynthetic process | 5.73E-04 |
| | | signaling | 9.36E-04 |
| | | nervous system development | 1.65E-03 |
| | | positive regulation of biological process | 1.72E-03 |
| | | regulation of response to stimulus | 3.22E-03 |
| | | response to stress | 5.78E-03 |
| | | tissue development | 7.57E-03 |
| | General | cellular catabolic process | 1.38E-05 |
| | | cellular component assembly at cellular level | 1.66E-04 |
| | | protein modification process | 5.73E-04 |
| | | establishment of localization in cell | 5.86E-04 |
| | | multicellular organismal reproductive process | 1.07E-03 |
| | | gene expression | 1.17E-03 |
| | | macromolecular complex subunit organization | 1.38E-03 |
| | | regulation of localization | 1.81E-03 |
| | | developmental process involved in reproduction | 2.14E-03 |
| | | organ morphogenesis | 2.23E-03 |
| | | regulation of protein metabolic process | 2.62E-03 |
| | | response to organic substance | 2.80E-03 |
| | | cell communication | 3.03E-03 |
| | | sexual reproduction | 3.22E-03 |
| | | regulation of catalytic activity | 3.22E-03 |
| BP | General | cell projection organization | 4.62E-03 |
| | | response to external stimulus | 4.84E-03 |
| | | membrane organization | 5.05E-03 |
| | | cell cycle | 5.68E-03 |
| | | protein localization | 5.78E-03 |
| | | homeostatic process | 5.97E-03 |
| | | protein complex biogenesis | 6.01E-03 |
| | | lipid metabolic process | 6.61E-03 |
| | | cellular macromolecule localization | 6.80E-03 |
| | | regulation of signal transduction | 7.59E-03 |
| | | intracellular signal transduction | 8.27E-03 |
| | | sensory organ development | 9.44E-03 |
| | | response to abiotic stimulus | 9.83E-03 |
| | Specific | alcohol metabolic process | 3.03E-03 |
| | | cellular macromolecule catabolic process | 8.59E-03 |
| | | proteolysis | 9.44E-03 |
| MF | Highly-general | catalytic activity | 1.56E-06 |
| | | protein binding | 6.79E-04 |
| CC | Highly-general | intracellular organelle part | 1.94E-11 |
| | | macromolecular complex | 7.28E-08 |
| | | cytoplasmic part | 1.02E-06 |
| | | intracellular membrane-bounded organelle | 2.71E-06 |
| | | membrane | 1.17E-03 |
| | | cell periphery | 6.13E-03 |
| | General | organelle membrane | 1.91E-03 |
| | | cytoskeletal part | 3.84E-03 |
| | | cell projection | 5.95E-03 |
| | | endomembrane system | 6.79E-03 |
| | | cell fraction | 7.71E-03 |
| | | nuclear lumen | 9.70E-03 |
| | Specific | ribonucleoprotein complex | 6.79E-03 |
| | | nucleoplasm part | 7.80E-03 |
