# Supplementary material for: A domain-centric solution to functional genomics via dcGO Predictor
Source: BMC Bioinformatics. 2013 Feb 28;14(Suppl 3):S9. doi: 10.1186/1471-2105-14-S3-S9 (PMC3584936; doi:10.1186/1471-2105-14-S3-S9)
Supplement: Additional file 4 — Enriched GO terms for domain repertoire present at each of three genomes of animal-fungal boundary. [file 1471-2105-14-S3-S9-S4.pdf]

Additional file 4. Enriched GO terms for domain repertoire present at each of three genomes of animal-fungal boundary.

Ontology	Level	GO Term	FDR
Capsaspora owczarzaki			
BP	Highly-general	primary metabolic process	5.29E-08
		small molecule metabolic process	1.06E-04
		nitrogen compound metabolic process	1.91E-04
		cellular macromolecule metabolic process	1.83E-03
	General	cellular catabolic process	1.91E-04
		heterocycle metabolic process	9.42E-04
		organic acid metabolic process	5.93E-03
		cellular ketone metabolic process	7.00E-03
MF	Highly-general	catalytic activity	9.87E-08
CC	Highly-general	intracellular organelle part	8.93E-07
		macromolecular complex	8.42E-05
		intracellular non-membrane-bounded organelle	3.68E-03
	General	mitochondrion	7.44E-03
Monosiga brevicollis			
BP	Highly-general	primary metabolic process	1.32E-08
		small molecule metabolic process	9.31E-06
		biosynthetic process	1.26E-04
		cellular component organization at cellular level	2.30E-04
		nitrogen compound metabolic process	4.07E-04
		cellular component biogenesis	1.95E-03
		localization	9.14E-03
	General	cellular catabolic process	2.30E-04
		organic acid metabolic process	1.95E-03
		nucleobase-containing small molecule metabolic process	2.43E-03
		small molecule catabolic process	4.67E-03
		cellular component assembly at cellular level	7.99E-03
		amine metabolic process	8.74E-03
MF	Highly-general	catalytic activity	2.56E-09
CC	Highly-general	intracellular organelle part	1.29E-08
		cytoplasmic part	1.70E-06
		macromolecular complex	2.11E-06
		intracellular membrane-bounded organelle	5.19E-06
		intracellular non-membrane-bounded organelle	7.86E-04
	General	organelle membrane	2.56E-03
		mitochondrion	3.85E-03
Proterospongia			
BP	Highly-general	primary metabolic process	1.60E-07
		small molecule metabolic process	1.86E-04
		biosynthetic process	3.66E-04
		cellular macromolecule metabolic process	1.46E-03
		nitrogen compound metabolic process	2.17E-03
	General	cellular catabolic process	1.23E-03
		organic acid metabolic process	5.84E-03
		heterocycle metabolic process	5.88E-03
MF	Highly-general	catalytic activity	1.46E-07
CC	Highly-general	intracellular organelle part	3.54E-08
		macromolecular complex	2.24E-05
		cytoplasmic part	4.40E-05
		intracellular membrane-bounded organelle	4.78E-05
		intracellular non-membrane-bounded organelle	4.35E-04
